# Ground-state orbital angular momentum lasing from liquid crystal torons embedded in a microcavity

**DOI:** 10.1126/sciadv.aeb6167

**Published:** 2026-03-13

**Authors:** Marcin Muszyński, Daniil Bobylev, Piotr Kapuściński, Przemysław Oliwa, Joanna Mędrzycka, Eva Oton, Rafał Mazur, Przemysław Morawiak, Wiktor Piecek, Przemysław Kula, Dmitry Solnyshkov, Guillaume Malpuech, Jacek Szczytko

**Affiliations:** ^1^ Institute of Experimental Physics, Faculty of Physics, University of Warsaw, Warsaw, Poland.; ^2^ Université Clermont Auvergne, Clermont Auvergne INP, CNRS, Institut Pascal, F-63000 Clermont-Ferrand, France.; ^3^ Institute of Applied Physics, Military University of Technology, Warsaw, Poland.; ^4^ Institute of Chemistry, Military University of Technology, Warsaw, Poland.; ^5^ Institut Universitaire de France (IUF), 75231 Paris, France.

## Abstract

Orbital angular momentum laser beams have many important practical applications, such as optical tweezers or ultrafast communications. Creating such beams represents an important challenge in photonics. Here, we demonstrate that torons, which are topological defects in liquid crystal textures, when embedded in a microcavity, generate a real-space non-Abelian gauge field responsible for the topological inversion of ground and excited states. The resulting ground state produces robust lasing with nonzero orbital angular momentum in each of the circular polarization components.

## Teaser

Torons, topological defects in liquid crystals, in a microcavity produce robust lasing with nonzero orbital angular momentum.

## INTRODUCTION

Optical vortices are topological phase singularities in electromagnetic waves (*1*). Although first noted in the 1950s (*2*–*4*) and described in 1974 (*5*), optical vortices were truly recognized in 1992 (*6*) when vortex beams were shown to carry a quantized orbital angular momentum (OAM). This discovery paved such promising routes in optical communication and optical manipulation (*7*) that vortex beams and OAM beams became synonymous albeit there is no general straightforward relation between phase vorticity and OAM (*8*). Typically, OAM-carrying vortex beams are non–ground-state solutions of the paraxial wave equation which are characterized by a phase factor *e*^*il*φ^ in the plane perpendicular to the beam axis, where φ is the polar angle and *l* ≠ 0 is an integer called a topological charge. Because of the emergent helical wavefronts, such beams carry an intrinsic (*9*) longitudinal (*10*) OAM equal to ℏ*l*. Owing to the similarity between the paraxial wave equation and the Schrödinger equation, these beams can be used to emulate vortex dynamics in quantum fluids of light (*11*). They have also been demonstrated to be a useful tool for quantum computing (*12*, *13*). OAM vortex beams can be produced using bulk optics methods to imprint the required phase distribution onto an incident beam (*14*). However, these approaches are neither scalable nor tunable, require extremely high fabrication precision and impose restrictions on the incident beam. As a result, many efforts are focused on producing on-chip OAM lasers (*15*). OAM can be coupled to another type of light angular momentum, spin angular momentum (SAM), which is associated with its polarization, resulting in spin-orbit coupling (SOC) of light (*16*, *17*). One strategy to realize on-chip OAM lasers has been based on etching planar semiconductor microcavities to fabricate photonic molecules with a ring topology. States with a given absolute value of OAM form a degenerate quadruplet because of the polarization (spin) degree of freedom. This degeneracy can be partially lifted by the SOC (*18*), and further fully removed by pump-induced spin-anisotropic interactions, allowing OAM lasing (*19*).

Among other techniques, nonzero OAM states can often be achieved with synthetic gauge fields, such as emergent magnetic fields with Landau levels (*20*). This is an active field of research, overlapping with the field of topological photonics (*15*), because many topologically protected states can also be interpreted using the gauge field formalism (*21*). SOC terms can sometimes be represented in the minimal coupling form, and thus they can be seen as gauge potentials (*22*). The resulting gauge fields are often non-Abelian, offering interesting perspectives going beyond the well-explored Abelian fields (*23*–*25*). The non-Abelian nature of such gauge fields makes them quite different from the electromagnetic field: not only a constant “vector potential” can correspond to a nonzero field (*26*) but also the “scalar potential” (not coupled to the momentum via minimal coupling) can give rise to nonzero angular momentum states, as we show below.

In this context, liquid crystals (LCs) appear as a very efficient resource: they were used to couple SAM to the extrinsic OAM in nematic LC microcavity resulting in electrically tunable giant Rashba-Dresselhaus SOC (*27*) and in cholesteric (chiral nematic) LC microcavities resulting in SOC photonic bands (*28*). LCs support a plethora of topological defects (*29*, *30*), which are used to couple SAM to the intrinsic (vortex-induced) OAM in q-plates (*31*), forked edge dislocations (*32*), and nematic droplets (*33*). Recently, the emission of laser vector beams (*34*), i.e., the beams with spatially dependent polarization and no OAM, from various self-assembled topological LC defects confined to a microcavity was demonstrated (*35*).

In this work, we report on the implementation and characterization of a self-assembled topological structure consisting of chiral LC (CLC) torons inside an optical microcavity exhibiting an unconventional ladder of spin-orbit coupled states. In notable contrast to the normal-state ordering, the ground state in our system has an optical vortex, which is confirmed by fork interference patterns in lasing experiment. We develop a theoretical model based on the effective stationary Schrödinger equations with the position-dependent term that couples different polarizations. It is equivalent to a real-space non-Abelian gauge field, driving the topological transition with the inversion of the state ordering. We further analyze the spectrum depending on the size of torons unveiling the topological nature of such mode order.

## RESULTS

**Fig. 1.**
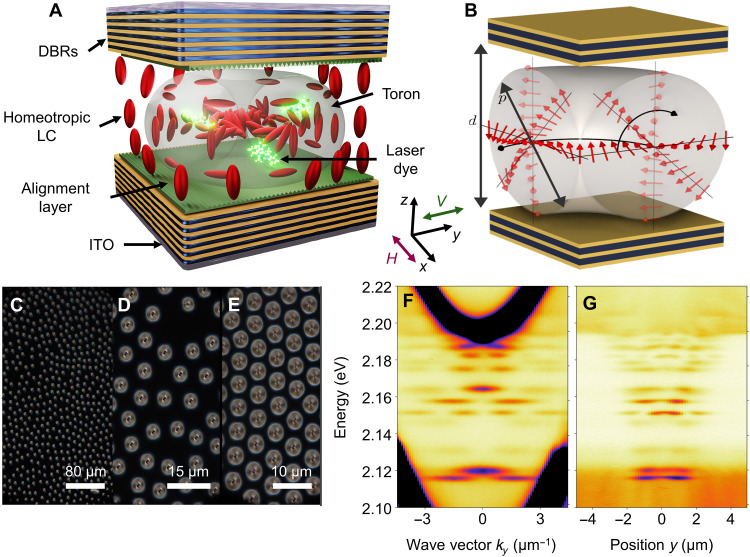
Chiral nematic LC torons. (**A**) Scheme of the dye-doped optical microcavity with an embedded toron structure. The vertical polarization (green double arrow) is defined along the *y* axis (parallel to the spectrometer slit), and the horizontal polarization (purple double arrow) along the *x* axis (perpendicular to the slit). (**B**) Sketch of the director distribution of an elementary toron. (**C** to **E**) Cross-polarized white light transmission images of torons for different film thicknesses *d* (local cavity thickness). (**F**) Momentum- and (**G**) spatially resolved transmission spectra of single toron confined within the LC microcavity.

The scheme of the investigated sample is presented in Fig. 1A. The microcavity, composed of two TiO_2_/SiO_2_-based distributed Bragg reflectors, hosts a homeotropically oriented LC matrix. In regions where the orienting layer imposes structural frustration, chiral topological defects, known as torons, spontaneously form. Figure 1B schematically illustrates the distribution of the LC director inside a toron. The toron size, filling factor, and spacing are influenced by both the elastic properties of the LC and the imposed boundary conditions, e.g., cavity thickness and orienting layer direction. The diameter of the toron (determined by the pitch *p* of the supramolecular helical structure) is constrained by the local cavity thickness *d*. This diameter can be dynamically tuned by applying an external electric field to the transparent indium tin oxide (ITO) electrodes, which leads to the toron compression. Furthermore, doping the LC mixture with the organic laser dye pyrromethene 580 (P580) enables the observation of optically pumped lasing carrying OAM.

Figure 1 (C to E) presents polarized optical microscopy images of three regions of the LC cell, showing both well-isolated, noninteracting torons and a packed triangular lattice. The first case configuration allows for the investigation of a single toron inside the microcavity. Figure 1 (F and G) shows momentum- and spatially resolved transmission spectra of a typical noninteracting toron with a diameter of 4 μm. The toron acts as a photonic trap, forming a binding potential and a series of localized optical states. However, unlike other photonic traps based on planar microcavities (micropillars and mesas), the ground state at 2.115 eV exhibits a minimum intensity at *k* = 0. Note that continuous spectral features above 2.195 eV and below 2.14 eV correspond to the continuum of states of subsequent cavity modes in the area surrounding the toron. For more information about photonic states in optical microcavities, see section S1.

**Fig. 2.**
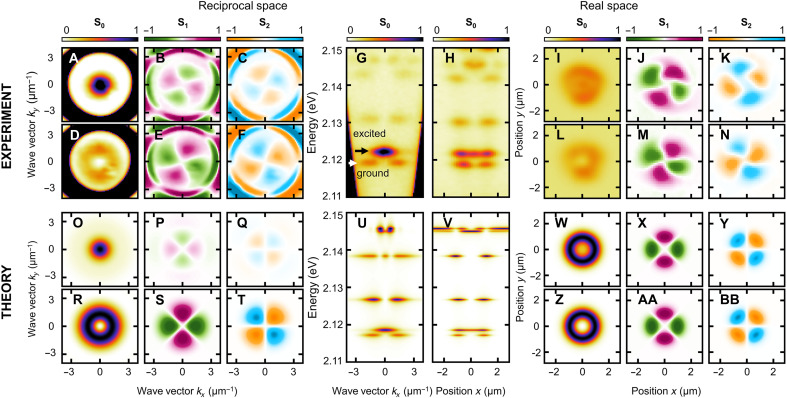
Spectral and polarization properties of photonic states bound by a single toron in a LC microcavity. Experimental results [(A) to (N)]: reciprocal space *S*_0,1,2_ Stokes parameters distributions corresponding to the first excited states (**A** to **C**) and to the ground state (**D** to **F**), respectively; momentum (**G**) and spatially (**H**) resolved transmission spectra with black and white arrows indicating the energies of the first excited and the ground states, respectively; real-space *S*_0,1,2_ Stokes parameters distributions corresponding to the first excited states (**I** to **K**) and to the ground state (**L** to **N**). Theoretical results [(O) to (BB)]: reciprocal space *S*_0,1,2_ Stokes parameters distributions corresponding to the first two degenerate excited states (**O** to **Q**) and to the ground state (**R** to **T**); reciprocal (**U**) and real (**V**) space intensity spectra along *k_y_* = 0 and *y* = 0, respectively; real-space *S*_0,1,2_ Stokes parameters distributions corresponding to the first two degenerate excited states (**W** to **Y**) and to the ground state (**Z** to **BB**).

To investigate this peculiar energy ladder, polarization-resolved tomography of both reciprocal and real-space transmissions of a single toron was performed. Figure 2 (A to C and D to F) presents the isoenergetic planes in reciprocal space for the Stokes parameters *S*_0_, *S*_1_, and *S*_2_ for the first excited state and the ground state, respectively. A comparison of the total intensity distributions (Fig. 2, A and D) reveals a key difference. While the ground state exhibits a radially symmetric intensity profile with a central minimum, the first excited state features a central maximum intensity at normal incidence, whereas the opposite would be expected qualitatively from the node theorem.

The saturated outer ring observed at wave vectors greater than 3 μm^−1^ is associated with transmission through planar cavity modes (regions outside of the toron), and therefore does not present any interest for our study. Because of the strong transverse electric–transverse magnetic splitting induced by the birefringence of the LC, the linear polarization pattern of the *S*_1_ component (Fig. 2, B and E) for these cavity modes forms a characteristic double quadrupole, which remains independent of energy. For localized states, the *S*_1_ distribution also forms a quadrupole pattern.

However, because of the rotating director of the LC molecules inside the toron and the resulting longitudinal-transverse (LT) splitting (longitudinal means along the circumference of the toron seen from above and transverse corresponds to the radial direction), this quadrupole is rotated with respect to those of the cavity modes. Furthermore, the polarization character of the two lowest-energy states is opposite (the quadrupoles are rotated by 90 degrees relative to each other). Complementary results are obtained for diagonal polarizations (Fig. 2, C and F), where the *S*_2_ quadrupole is rotated by 45° relative to *S*_1_. The real-space distribution of the Stokes components is shown in Fig. 2 (I to N). The intensity profiles for both lowest-energy states (Fig. 2, I and L) exhibit a radially symmetric donut shape, with an additional central maximum for the excited state. Similar to reciprocal space, the linear polarization patterns of both states form quadrupoles (Fig. 2, J, K, M, and N).

To get qualitative insights into the observed properties, we use a theoretical model based on the effective two-dimensional (2D) stationary Schrödinger equations (the derivation and calculation details are provided in the “Theoretical models and numerical simulations” section in Materials and Methods). In short, a Fabry-Perot microcavity filled with a LC is naturally described by 3D Maxwell’s equations in an inhomogeneous anisotropic dielectric media. The permittivity tensor is defined by the LC director, a vectorial order parameter that determines the averaged local orientation of the LC molecules. The director configuration of a toron can be represented by a double-twist cylinder bent into a torus possessing Hopf fibration features (*30*) (see Fig. 1). This problem without additional simplifications is cumbersome even for a full-wave numerical simulation. To simplify the treatment, we note that at optical frequencies the cavity mode numbers are relatively high (*N* > 10), so the paraxial approximation can be used. In addition, the electromagnetic field is strongly confined to the emergent optical trap, so the slowly varying medium approximation can be used. These assumptions allow us to neglect the out-of-plane electric field component and choose only one cross section of the 3D toron that couples the in-plane electric field components, the equatorial cross-section represented by a skyrmion. Ultimately, we end up with a system of two Schrödinger equations in two spatial dimensions, where the effective mass is due to the microcavity photon dispersion and the two components of the wave function correspond to horizontal and vertical polarization states. Beside the kinetic energy and the potential terms, there are also terms proportional to the $\hat{\sigma }$_1_ and $\hat{\sigma }$_3_ Pauli matrices (in the chosen linear polarization basis), which correspond to the texture of spatially dependent LT splitting. These equations define the eigenmodes which are calculated numerically using realistic parameters. Figure 2 (O to Q and R to T) depicts the retrieved Stokes polarization parameter distributions in reciprocal space. The obtained polarization textures of the localized modes correspond to the experimental ones. Figure 2 (U and V) shows the retrieved spectra obtained by summing the intensity contributions from the *k_y_* = 0 cross-sections (in reciprocal space) and *y* = 0 cross sections (in real space) from individual eigenmodes. Figure 2 (W to Y and Z to BB) depicts the retrieved Stokes polarization parameters distributions in real space. Again, the obtained polarization textures coincide with those observed experimentally.

Thus, the observed polarization and spectral properties can be qualitatively understood by considering a spatially dependent texture of the LT splitting. It should be noted that despite its simplicity, our model reproduces the experimental results remarkably well and requires much less computational resources than full-wave 3D numerical simulations.

**Fig. 3.**
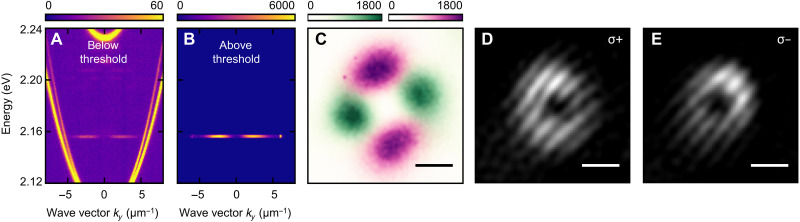
Lasing with OAM. Momentum-resolved photoluminescence spectra measured (**A**) below and (**B**) above the lasing threshold. (**C**) Real-space image of the overlapped horizontally *H* (pink) and vertically *V* (green) polarized lasing signals from a toron. (**D** and **E**) Interference patterns collected for σ^+^ and σ^−^ polarizations, respectively. The fork-like patterns with opposite orientations indicate the presence of OAM with opposite signs for the two circular polarization states. Scale bars, 1.5 μm.

A quadrupolar polarization distribution is often a signature of phase singularities underlying OAM (*18*, *19*). The LC mixture used in the sample is doped by a laser dye, which allows lasing to be achieved from a single toron state and the investigation of its phase properties. Figure 3 (A and B) shows the representative momentum-resolved photoluminescence spectra of a single toron of 4.2 μm diameter below and above the lasing threshold, respectively (see fig. S1 for intensity and linewidth as a function of excitation pulse energy). Below the threshold, the strongest signal originates from microcavity modes in the region surrounding the toron. The spectrum has been partially saturated to reveal weaker spontaneous emission from several toron states, including the ground state at approximately 2.16 eV. Above the threshold, the emission signal is dominated by the ground-state lasing emission with the minimum intensity at *k* = 0. The superimposed lasing signals from multiple noninteracting torons in the real space (Fig. 3C) with horizontal (pink) and vertical (green) polarization form a quadrupole pattern rotated with respect to the *XY* axis, consistent with the transmission measurements in Fig. 2M. Notably, the intensity profile of each toron follows the shape of the ground state, exhibiting a minimum in the center, a characteristic feature of OAM-carrying light. To further verify the presence of phase singularities, interferometric measurements of the lasing signal from a single toron were performed in σ^+^ and σ^−^ polarizations (Fig. 3, D and E). The resulting fork-like interference patterns, which are mirrored for opposite polarizations, confirm (*19*) that lasing from the lowest state carries OAM with opposite signs in two orthogonal circular polarizations.

**Fig. 4.**
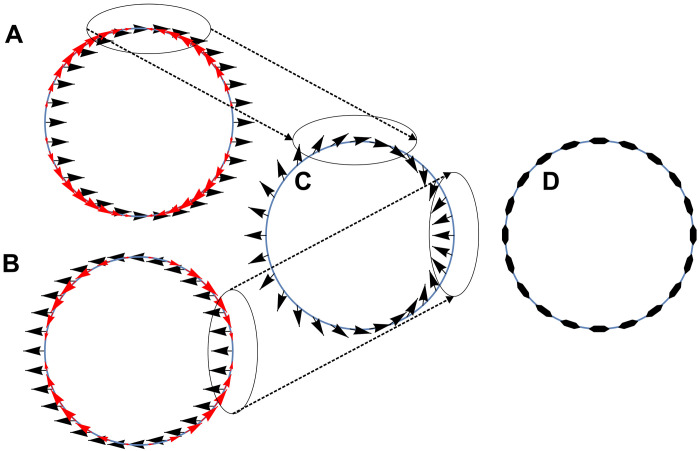
Non-Abelian gauge field effect. (**A** and **B**) Uniform Stokes vector distribution (black arrows) of the H/V-polarized (respectively A/B) ordinary (zero OAM) ground state and the tangential force acting on it (red arrows). (**C**) Resulting Stokes vector distribution (black arrows) after the action of the force. Ellipses mark the regions where the corresponding spin is concentrated by the force. (**D**) The distribution of the linear polarization (double-headed arrows) corresponding to the Stokes vector shown in (C).

The nonzero OAM property of the ground state can also be interpreted from the point of view of a classical non-Abelian gauge field theory (*22*). The spatially dependent birefringence can be seen as a varying non-Abelian gauge potential, giving rise to a gauge field, acting on the spin distribution similarly to the Lorentz force acting on electromagnetic charges. It leads to the redistribution of the spin along the toron (see Fig. 4) and to the topological inversion of the state ordering (the details are provided in the “Theoretical models and numerical simulations” section in Materials and Methods). This allows us to conclude that the OAM states observed in our system are similar to Landau levels stemming from a non-Abelian equivalent of a magnetic field.

**Fig. 5.**
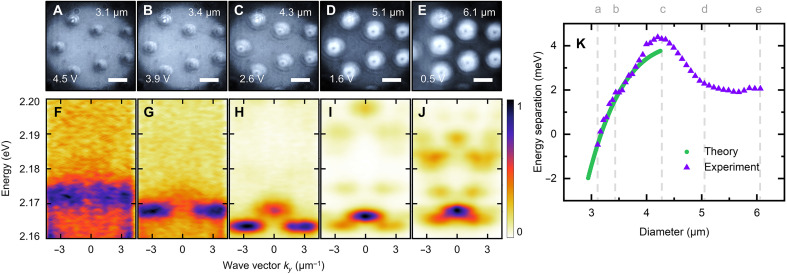
Voltage tunability of the photonic trap size. (**A** and **E**) Real-space transmission images of multiple torons recorded at different applied voltages (indicated in the bottom-left). The estimated toron diameters are labeled in the top-right and the scale bar is 5 μm. (**F** to **J**) Normalized momentum-resolved transmission spectra corresponding to the applied voltages in (A) to (E). (**K**) Experimental (purple triangles) and calculated (green circles) energy separation between ground and excited states. Dashed lines correspond to the toron diameters shown in (A) to (E).

The LC medium provides remarkable tunability of the photonic modes and the photonic band structure (*27*, *28*). We use this property to dynamically control the size of the toron and its energy spectrum. Figure 5 (A to E) presents real-space transmission images of several torons under different applied voltages, showing a gradual increase in diameter from approximately 3.1 to 6.1 μm as the voltage decreases. While positions of the torons remain mostly fixed, occasional lateral shifts are observed (e.g., the leftmost toron in Fig. 5E), but most remain unaffected.

This size dependence strongly affects the energy spectrum of the torons, as seen in the momentum-resolved transmission spectra (Fig. 5, F to J). For the smaller torons, only the two lowest-energy states can be observed, with nearly degenerate energies. We plot the energy separation between these states, extracted from the Lorentzian curve fitting (for details, see the “Measurements” section in Materials and Methods), in Fig. 5K. For the smallest toron size, the states exhibit “normal” ordering: The lowest energy state has zero OAM in both spin components. For higher sizes, a topological transition with the inversion of the state ordering (*36*–*38*) is observed: The lowest energy state is now the one with nonzero OAM, opposite in two spin components. It is in this topologically nontrivial regime that all the observations reported above were made. It should be emphasized that reducing the toron size does not enable switching of the lasing between the nontrivial and trivial states, because this process simultaneously lowers the *Q*-factor of both modes. Consequently, lasing from the toron states vanishes before the topological transition occurs, and instead emerges from the surrounding microcavity mode (see fig. S2).

To validate the emergence of the size-driven topological transition theoretically, we compute the three lowest energy eigenmodes of the derived effective Schrödinger equations for different toron radii. The obtained spectral separation between the ground state and the excited states as a function of the toron radius is shown in Fig. 5K. The model demonstrates remarkable agreement with the experimental results, especially in the transition region. For larger torons, the theory deviates from the experiment because of an approximate representation of the birefringence profile (see Materials and Methods for more comments).

## DISCUSSION

We have demonstrated OAM lasing from topological defects (torons) in LC microcavities and revealed a topological transition associated with the exchange of the ground and excited states in these torons. More fundamentally, we show that spatially varying birefringence of the LC texture can be seen as a non-Abelian gauge field leading to the inversion of the state ordering and responsible for the ensemble of the observations. Our study opens a broad avenue for the studies of the fundamental effects and potential applications of non-Abelian gauge fields in photonic systems

## MATERIALS AND METHODS

### Sample preparation

The distributed Bragg reflectors (DBRs) consisted of six TiO_2_/SiO_2_ pairs centered at 550 nm (2.25 eV) and were deposited on ITO-coated glass substrates. The desired cavity thicknesses were obtained using calibrated silica spacers. In photoluminescence measurements, the *Q*-factor of the lasing mode below threshold was approximately 1150, slightly higher than that of the trivial mode (typically below 1000).

Several methods, including electric field–induced unwinding (*39*), localized laser perturbation (*40*), and thermal quenching from the isotropic phase (*41*, *42*) can generate torons. In the latter case, rapid cooling promotes the spontaneous nucleation of topological solitons as the chiral nematic order emerges, with the cooling rate controlling the number and size of torons formed. This thermal-quench mechanism has been experimentally demonstrated in recent works (*41*, *42*) and provides a robust, reproducible route to generating stable defect configurations in confined cholesteric systems. We use this method to nucleate torons under homeotropic conditions. First, it is necessary to align the helical axis parallel to the substrate. Surface treatments that support uniform homeotropic alignment (LC molecules perpendicular to the substrates) can induce this structure. The homeotropic anchoring was achieved using a SE-4811 polyimide alignment layer (Nissan Chemicals), spin-coated on DBRs and cured following the manufacturer’s protocol. This polyimide provides strong, uniform vertical anchoring, which is essential for stabilizing the confined cholesteric defect structures and ensuring reproducible nucleation of torons under our experimental conditions. However, the chiral dopant that is added to the precursor LC mixture must be sufficient for the CLC to maintain its chirality in the microcavity. The minimum helical pitch length *p_*th*_* to achieve a homeotropic state can be expressed as

$$p_{\mathrm{th}}=2d\frac{K_{2}}{K_{3}}$$ (1)

where $K_{2}$ and $K_{3}$ are the twist and bend elastic constants of the LC, respectively and d is the cell thickness. If the pitch length is longer than $p_{\mathrm{th}}$, the chiral torque is weak, the LCs will not twist, and the LC molecules will align homeotropically. If the pitch length is shorter than $p_{\mathrm{th}}$, there is enough chiral torque and different chiral structures are formed in the microcavity. The stabilization of a CLC structure can become quite complex if the CLC helices need to be homogeneously aligned and stable over time. Here, for stabilization, the tempering method was used.

Tempering involves introducing the LC material inside the cavities in the isotropic state (temperature above the LCline phase). The cavity then undergoes a temperature process in which it is rapidly cooled to less than 4°C and then slowly heated to room temperature. LC flow and rapid turbulence allow for the formation of stable and well-defined torons.

### Measurements

The optical measurements are performed at room temperature. The scheme of the experimental setup is shown in fig. S3. The microscope objective with 60× magnification, numerical aperture (NA) = 0.7, and coverglass correction ring (Nikon CFI S Plan Fluor ELWD 60XC) is used for the collection of light. An additional microscope objective with 20× magnification and NA = 0.4 is used for excitation (Nikon CFI TU Plan Epi ELWD 20X). The set of a quarter-wave plate, a half-wave plate, and a linear polarizer is used to collect light in the selected polarization state. The collected signal is spectrally dispersed using a Czerny-Turner Shamrock 750 spectrograph and subsequently recorded by a charge-coupled device camera (Andor Newton DU940P). Depending on whether reciprocal space (*k*-space) or real space (*r*-space) is imaged on the camera, either a pair of lenses or a single lens is placed in the optical path, mounted on motorized translation stages, which allows for tomography measurements. An additional camera, equipped with a Wollaston prism, enables simultaneous measurement of two orthogonal linear polarizations. Furthermore, the introduction of an interferometer in a Mach-Zehnder configuration with a removable quarter-wave plate, a half-wave plate, and a linear polarizer in this beam path facilitates polarized interference measurements. For emission measurements, the sample is excited by a Q-switched diode-pumped laser with 100 pulses (Cobolt Tor XS, 532-nm center wavelength, 2-ns pulse duration). For transmission measurements, the sample is illuminated with a white light source equipped with an additional band-pass filter to minimize sample heating. The excitation spot diameter is approximately 10 μm for photoluminescence measurements, and approximately 30 μm for transmission measurements. To tune the lateral size of torons, the LC microcavity is addressed by an AC waveform generator (Rigol DG2041A) with applying a 50 Hz square signal and varying amplitude ranging from 0.1 to 5.4 V.

To determine the energies of the ground and excited states (Fig. 5), the momentum-resolved spectra were integrated over two separate wave vector ranges: for the excited state, between 0 ± 0.5 μm^−1^; and for the ground state, around 2.75 ± 0.5 μm^−1^. Within these wave vector ranges the states do not overlap, which allows each spectrum to be fitted with a single Lorentzian profile and the energies of both states to be extracted independently.

### Theoretical models and numerical simulations

#### Schrödinger formalism

The effective stationary Schrödinger equations used for the qualitative theoretical analysis of the considered system are derived from the time-harmonic ($e^{-i\omega t}$) Maxwell’s equations in an inhomogeneous anisotropic dielectric assuming the slowly varying medium and paraxial approximations. Specifically, the wave equation for the electric field

$$\nabla \times \nabla \times E-\frac{\omega^{2}}{c^{2}}\hat{\varepsilon }\left(r\right)E=0$$ (2)

with the permittivity tensor $\hat{\varepsilon }$(**r**) defined by its principal values $\varepsilon_{o,e}\;\left(\Delta \varepsilon =\varepsilon_{e}-\varepsilon_{o}>0\right)$ and the LC director **d**(**r**) as ε*_ij_* = ε_o_ δ*_ij_* + ∆ε *d_i_d_j_*, can be transformed into the system of two coupled Schrödinger equations

$$\begin{array}{c} \left\{\left[\frac{-\hbar^{2}}{2m}\nabla^{2}-\frac{\hbar \omega }{2}\left(\varepsilon_{o}+\Delta \varepsilon \frac{d_{x}^{2}+d_{y}^{2}}{2}\right)\right]{\hat{\sigma }}_{0}-\frac{\hbar \omega }{2}\Delta \varepsilon d_{x}d_{y}{\hat{\sigma }}_{1}\right.\\ \left.-\frac{\hbar \omega }{2}\left(\Delta \varepsilon \frac{d_{x}^{2}-d_{y}^{2}}{2}\right){\hat{\sigma }}_{3}\right\}\psi =\frac{-\hbar^{2}k_{z}^{2}}{2m}\psi \end{array}$$ (3)

provided $E\left(x,y,z\right)\approx {\left[\psi_{x}\left(x,y\right),\psi_{y}\left(x,y\right),0\right]}^{T}\;e^{-ik_{z}z}=\psi \;e^{-ik_{z}z}$ and ∇(∇·E)≈0. The latter conditions hold when considering large cavity mode numbers and strong confinement within the optical trap, assuming the *z* axis is perpendicular to the cavity mirrors. By *m* = (ℏω)/(*c*^2^) and σ_0,1,2,3_, we denote the effective photon mass and the identity/Pauli matrices, respectively. The director field components $d_{x}=-d_{\varphi }\;\text{sin}\left(\varphi \right)$, $d_{y}=d_{\varphi }\;\text{cos}\left(\varphi \right)$, where *d*_φ_ = sin(*q*ρ) for $\rho \leq r_{0}$ and $d_{\varphi }=0$ for $\rho >r_{0}$ [as usual, $\rho =\sqrt{x^{2}+y^{2}}$ and $\varphi ={\text{atan}}_{2}\left(y,x\right)$], correspond to a skyrmionic equatorial cross section of the toron of radius $r_{0}\left(q=\pi /r_{0}\right)$, which encodes the necessary symmetries to couple the in-plane electric field components.

The derived system is solved numerically using the Schrödinger equation physics interface of COMSOL Multiphysics simulation software. The angular frequency ω = 2π*c*/λ corresponds to the vacuum wavelength λ = 589 nm of the experimentally observed lowest energy eigenmode; ordinary and extraordinary permittivity tensor components ε_o_ = 2.6179 and ε_e_ = 2.7357 are adjusted to ensure the topological transition at the experimentally observable toron size; the toron size *r*_0_ = 2 μm (Fig. 2) or variable (Fig. 5). The computational domain consists of a square with side length of 5*r*_0_ with zero probability boundary condition applied to all external boundaries. The eigenmodes are calculated using ARPACK algorithm searching for 16 eigenenergies. The obtained eigenmodes are filtered manually by inspecting the corresponding Stokes polarization parameters $S_{0}={|\psi_{x}|}^{2}+{|\psi_{y}|}^{2}$, $S_{1}={|\psi_{x}|}^{2}-{|\psi_{y}|}^{2}$, and $S_{2}={\left|1/\sqrt{2}\;\left(\psi_{x}+\psi_{y}\right)\right|}^{2}-{\left|1/\sqrt{2}\;\left(\psi_{x}-\psi_{y}\right)\right|}^{2}$ distributions in real and reciprocal spaces normalized to the maximal intensity *S*_0_. The *S*_3_ component of the Stokes vector, corresponding to the circular polarization degree, is zero.

We are using an approximated director profile for our simulations (a more exact one would require minimizing the elastic energy). For small toron sizes, the details of the refractive index profile do not play an important role: the amplitude of the electric field cannot change sufficiently rapidly in space to reflect the properties of the potential. This can be quantified by the following criterion: $r\ll \hbar /\sqrt{2\mathrm{mE}}$, where *E* is the quantization energy. In this case, the approximate theory gives results that are quite close to the experiment because they are profile independent. For larger torons, this does not hold anymore, and the differences become more important. The main reason why the separation between the modes becomes constant in experiment is that the overlap of the electric field with the region of in-plane director orientation becomes constant, whereas for smaller torons, an important part of the field remains outside of this region and changes with the toron size.

#### Non-Abelian gauge field

Gauge fields were introduced in physics by analogy with the electromagnetic field, and we will follow the same lines in our discussion. Our starting point is the Hamiltonian of a charged particle in an electromagnetic field, which reads

$$H=\frac{1}{2m}{\left(p-qA\right)}^{2}+\mathrm{qV}$$ (4)

where *q* is the charge of the particle (a scalar). The gauge potentials are: **A** (vector potential) and *V* (scalar potential).

The Yang-Mills field is a generalization of this description to a vectorial charge whose orientation is encoded in the components of the wave function. Initially, this vectorial charge was supposed to be the isospin that distinguishes a neutron and a proton

$$H=\frac{1}{2m}{\left(p-\eta A^{\left(a\right)}\sigma^{\left(a\right)}\right)}^{2}+\eta A_{0}^{\left(a\right)}\sigma^{\left(a\right)}$$ (5)

where η is the coupling constant (analog of the electron charge), **A**^(*a*)^ are the components of the analog of the vector potential, and σ^(*a*)^ are the generators of the SU(2) group (Pauli matrices). The non-Abelian behavior comes from the noncommutativity of these matrices. We note that this formula uses the Einstein summation convention (there is a sum over the repeating index *a*).

This non-Abelian gauge field became popular in spintronics and then in photonics, when it was understood (*22*, *43*) that the typical cases of SOC (such as the Rashba and Dresselhaus SOCs) can be described using this formalism. It applies to some photonic configurations as well (*26*, *44*, *45*). However, the Yang-Mills formalism is not limited to SOC, and it can describe various effective magnetic fields that are not coupled to the momentum (such as Zeeman splitting or linear birefringence). The most general form of the Schrödinger equation with the Yang-Mills gauge field is

$$i\hbar \frac{\partial \psi }{\partial t}=\left[\frac{1}{2m}{\left(p-\eta A^{\left(a\right)}\sigma^{\left(a\right)}\right)}^{2}+\eta A_{0}^{\left(a\right)}\sigma^{\left(a\right)}\right]\psi$$ (6)

For example, the Rashba term $\alpha \left(p_{x}\sigma^{\left(2\right)}+p_{y}\sigma^{\left(1\right)}\right)$ is represented by $A_{y}^{\left(1\right)}=A_{x}^{\left(2\right)}=2m\alpha ∕\left(\eta \hbar \right)$. In the present work, we focus on the representation of the Zeeman-like terms corresponding to the birefringence. They are encoded in the components of $A_{0}^{\left(a\right)}:a=0$ corresponds to the ordinary quantum-mechanical potential *U*(*x,y*), and *a* = 1 and *a* = 2 correspond to the energy splittings between horizontal/vertical and diagonal/antidiagonal polarizations, respectively. All these components of the non-Abelian gauge potential depend on the coordinates (*x,y*).

To calculate the force created by the emergent non-Abelian gauge field, we first rewrite the effective Schrödinger equations in circular basis (spinor form)

$$\left\{\frac{{\hat{p}}^{2}}{2m}+{\hat{V}}_{0}+\eta A_{t}^{\left(0\right)}{\hat{\sigma }}_{0}+\eta A_{t}^{\left(1\right)}{\hat{\sigma }}_{1}+\eta A_{t}^{\left(2\right)}{\hat{\sigma }}_{2}\right\}\psi =\frac{-\hbar^{2}k_{z}^{2}}{2m}\psi$$ (7)

where $\hat{p}=\left(-i\hbar \nabla \right){\hat{\sigma }}_{0}$, η=ℏ/2, ${\hat{V}}_{0}=\left(-\eta \omega \varepsilon_{\left(o\right)}\right){\hat{\sigma }}_{0}$, $A_{t}^{\left(0\right)}=-\omega \Delta \varepsilon \left(d_{x}^{2}+d_{y}^{2}\right)/2$, $A_{t}^{\left(1\right)}=-\omega \Delta \varepsilon \;\left(d_{x}^{2}-d_{y}^{2}\right)/2$, $A_{t}^{\left(2\right)}=-\omega \Delta \varepsilon \;d_{x}d_{y}$, $A_{t}^{\left(3\right)}=0$, and $\psi =1/\sqrt{2}\left(E_{x}+iE_{y},E_{x}-iE_{y}\right)$, and note that $A_{t}^{\left(i\right)}=\left(A_{t}^{\left(0\right)},A_{t}^{\left(1\right)},A_{t}^{\left(2\right)},A_{t}^{\left(3\right)}\right)$ can be viewed as a relativistic temporal part of a vector potential of a non-Abelian (Yang-Mills) gauge field (*22*). Spatial components of this vector potential (which are zero in our present case) would correspond to SOC terms of the Rashba-Dresselhaus type (*26*, *39*). At a given radius ρ, the link between the director field $\left(d_{x},d_{y}\right)$ and the coordinates (x,y) is given by

$$d_{x}=-\frac{y}{\rho }\text{sin}q\rho$$ (8)

$$d_{y}=\frac{x}{\rho }\text{sin}q\rho$$ (9)

The inner part of the toron that we consider corresponds to qρ<π, and therefore we have $d_{x}∼-y,d_{y}∼x$. The vector potential components become

$$A_{t}^{\left(1\right)}=\frac{\omega \Delta \varepsilon {\text{sin}}^{2}q\rho }{2\rho^{2}}\left(x^{2}-y^{2}\right)$$ (10)

$$A_{t}^{\left(2\right)}=\frac{\omega \Delta \varepsilon {\text{sin}}^{2}q\rho }{2\rho^{2}}2\mathrm{xy}$$ (11)

According to the Yang-Mills gauge field theory, the corresponding field tensor is

$$F_{\mu v}^{\left(i\right)}=\partial_{\mu }A_{v}^{\left(i\right)}-\partial_{\nu }A_{\mu }^{\left(i\right)}-\eta \epsilon^{\mathrm{ijk}}A_{\mu }^{\left(j\right)}A_{\nu }^{\left(k\right)}$$

Considering the Stokes polarization parameters as the time component of the spin four-current $J_{t}^{\left(i\right)}=\eta \left(S_{0},S_{1},S_{2},S_{3}\right)$, we recover the components of the non-Abelian analog of the Lorentz force

$$f_{\nu }=J_{\mu }^{\left(i\right)}F_{\mu \nu }^{\left(i\right)}$$ (12)

to be $f_{x}=-\zeta \left(xS_{1}+yS_{2}\right)$ and $f_{y}=\zeta \left(yS_{1}-xS_{2}\right)$, where $\zeta =\eta \omega ∆\varepsilon {\text{sin}}^{2}\left(q\rho \right)/\rho^{2}$. This coefficient depends on the radius ρ, but does not change sign within the toron.

To understand the effect of this non-Abelian analog of the Lorentz force, we consider its action on a state corresponding to the ground state of the system under “usual” state ordering (that is, with zero OAM and a uniform Stokes vector orientation). Two possible configurations are shown in Fig. 4 (A and B) with black arrows. They correspond to uniform horizontal (A) and vertical (B) polarizations. Considering that the particles are constrained to the ring-like trap, we compute only the tangential projection of the force $f_{\theta }=\left(f\cdot e_{\theta }\right)$. It is shown in panels (A) and (B) with red arrows. This force leads therefore to the redistribution of the particle density with the corresponding Stokes vector orientation (polarization), shown with black ellipses in panels (A) to (C) and the dashed lines connecting them: positive *S*_1_ (*H* polarization) accumulates along the vertical axis of the ring, while negative *S*_1_ (*V* polarization) accumulates along the horizontal axis. Similar results can be obtained for any Stokes vector orientation. The resulting Stokes vector distribution is shown in panel (C) and the corresponding polarization orientation appears in panel (D). It demonstrates that the non-Abelian force is the driving force behind the transition which makes the longitudinally polarized state (whose polarization is everywhere perpendicular to the radial direction, as seen in panel D) the ground state of the system.

## Supplementary Materials

### This PDF file includes:

Supplementary Text Figs. S1 to S3 References
