# Supplementary material for: Ground-state orbital angular momentum lasing from liquid crystal torons embedded in a microcavity
Source: Sci Adv. 2026 Mar 13;12(11):eaeb6167. doi: 10.1126/sciadv.aeb6167 (PMC12985666; doi:10.1126/sciadv.aeb6167)
Supplement: Supplementary file 1 — Supplementary Text Figs. S1 to S3 References [file sciadv.aeb6167_sm.pdf]

Science Advances
AAAS

Supplementary Materials for

Ground-state orbital angular momentum lasing from liquid crystal torons embedded in a microcavity

Marcin Muszyński *et al.*

Corresponding author: Guillaume Malpuech, guillaume.malpuech@uca.fr;
Jacek Szczytko, jacek.szczytko@fuw.edu.pl

Sci. Adv. **12**, eaeb6167 (2026)
DOI: 10.1126/sciadv.aeb6167

This PDF file includes:

Supplementary Text
Figs. S1 to S3
References

I. RESONANT STATES IN OPTICAL MICROCAVITIES

To provide the reader with a clearer understanding of the optical states in a microcavity with an embedded toron, where spatially dependent optical anisotropy is combined with a modulated photonic potential, we present here an extended background on optical resonances in related but simpler microcavity structures.

In a translationally invariant microcavity (i.e., without spatial modulation) filled with a birefringent nematic liquid crystal, two sets of optical modes with continuous parabolic dispersion and orthogonal linear polarizations emerge. When two modes of opposite parity are tuned into resonance, they become separated in momentum space and obtain a pronounced degree of circular polarization. This effect is known as emergent optical activity or Rashba-Dresselhaus spin-orbit coupling (*27*).

In the twisted-nematic phase, such coupled modes additionally acquire a nonzero photonic Berry curvature, which can be tuned electrically (*46*). In contrast, in a uniform lying helix, where the refractive-index modulation appears in the plane of the cavity and the cholesteric pitch is comparable to the optical wavelength, the system exhibits not only chirality but also a periodic photonic potential, giving rise to Bloch bands (*28*). When the molecular helix is tilted, these bands can further couple through interband spin-orbit interaction.

Spatial modulation without birefringence has historically been realized in many microcavities without liquid crystals, where structures of various geometries have been studied (*47*). Regions of higher refractive index act as local traps that host ladders of confined photonic states. These localized states can couple to form photonic "molecules" (*18*) and extended photonic "lattices" (*48*). Similar physics can also be explored in microcavities with tunable birefringence, which enables controlled photon tunneling between coupled sites (*49*).

Another key ingredient, present in essentially all microcavities, is the TE-TM splitting. Although the TE and TM modes are degenerate at $k_x = k_y = 0$, their different effective masses arise from the polarization-dependent reflection and transmission at each interface. This constitutes an intrinsic form of spin-orbit coupling. Its interplay with birefringence can give rise to synthetic gauge fields for photons (*44, 45*).

II. EXPERIMENTAL DETAILS

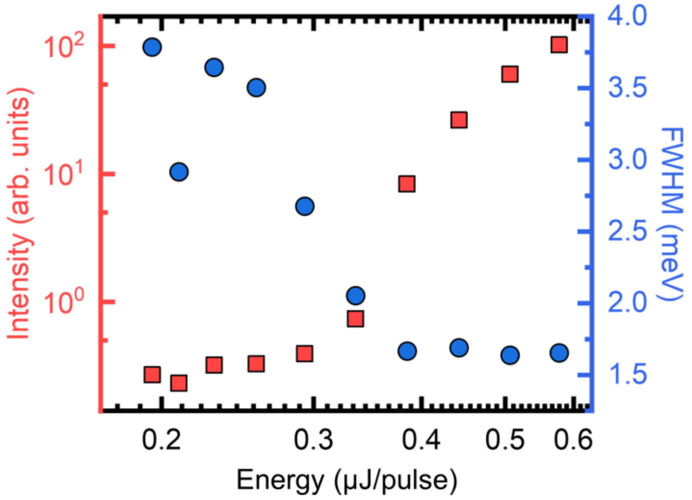

FIG. S1. **Ground-State Lasing of Toron-LCMC**. Emission peak intensity (red squares, logarithmic scale) and spectral linewidth (blue circles) as a function of excitation pulse energy, extracted by Lorentzian fitting.

Figure S1 presents the total intensity and linewidth of the ground state emission from a single toron as a function of the excitation pulse energy. A clear threshold behavior is observed at approximately 0.33 μJ/pulse, characterized by a nonlinear increase in emission intensity and a narrowing of the emission linewidth, confirming lasing.

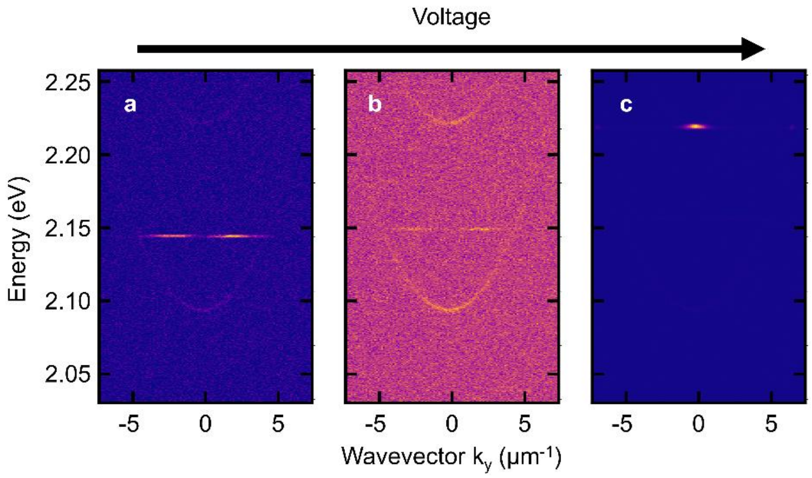

FIG. S2. **Momentum-resolved photoluminescence spectra collected at an excitation energy near the initial lasing threshold for different applied voltages: (a) 2 V, (b) 3 V, and (c) 4 V.**

Figure S2 presents momentum-resolved photoluminescence spectra collected at an excitation energy close to the initial lasing threshold for different applied voltages. At low voltage (Fig. S2(a)), lasing occurs from the non-trivial ground state, consistent with Fig. 3(b) in the main text. Upon increasing the voltage (Fig. S2(b)), this lasing is progressively suppressed. At even higher voltage (Fig. S2(c)), lasing reappears, but now from the surrounding microcavity mode rather than from the toron states.

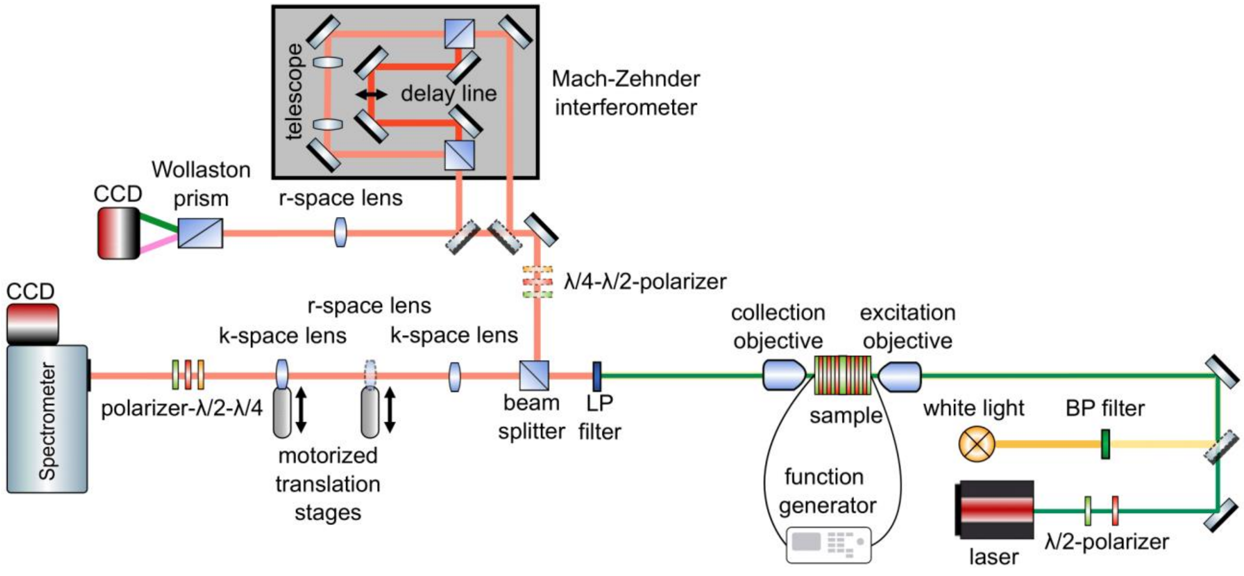

FIG. S3. **Scheme of the experimental setup.**

The experimental setup, described in detail in the Methods section of the main text, is shown in Figure S3. Optical paths are schematically indicated by colored lines: green for the excitation laser beam, yellow for the white light source, and red for the detected signal, which is split by a Wollaston prism into two linearly polarized components.

REFERENCES

1. P. Coullet, L. Gil, F. Rocca, Optical vortices. *Opt. Commun.***73**, 403–408 (1989).

2. H. Wolter, Zur frage des lichtweges bei totalreflexion. *Z. Naturforsch. A* **5**, 276–283 (1950).

3. J. Findlay, The phase and group paths of radio waves returned from region E of the ionosphere. *J. Atmos. Terres.* **1**, 353–366 (1951).

4. W. Braunbek, G. Laukien, Features of refraction by a semi-plane. *Optik* **9**, 174 (1952).

5. J. Nye, M. Berry, Dislocations in wave trains. *Proc. R. Soc. Lond. A* **336**, 165–190 (1974).

6. L. Allen, M. W. Beijersbergen, R. J. C. Spreeuw, J. P. Woerdman, Orbital angular momentum of light and the transformation of Laguerre-Gaussian laser modes. *Phys. Rev. A* **45**, 8185 (1992).

7. Y. Shen, X. Wang, Z. Xie, C. Min, X. Fu, Q. Liu, M. Gong, X. Yuan, Optical vortices 30 years on: OAM manipulation from topological charge to multiple singularities. *Light Sci. Appl.* **8**, 90 (2019).

8. M. V. Berry, W. Liu, No general relation between phase vortices and orbital angular momentum. *J. Phys. A Math. Theor.* **55**, 374001 (2022).

9. A. T. O’Neil, I. MacVicar, L. Allen, M. J. Padgett, Intrinsic and extrinsic nature of the orbital angular momentum of a light beam. *Phys. Rev. Lett.* **88**, 053601 (2002).

10. K. Y. Bliokh, F. Nori, Transverse and longitudinal angular momenta of light. *Phys. Rep.* **592**, 1–38 (2015).

11. I. Carusotto, C. Ciuti, Quantum fluids of light. *Rev. Mod. Phys.* **85**, 299 (2013).

12. F. Cardano, F. Massa, H. Qassim, E. Karimi, S. Slussarenko, D. Paparo, C. de Lisio, F. Sciarrino, E. Santamato, R. W. Boyd, L. Marrucci, Quantum walks and wavepacket dynamics on a lattice with twisted photons. *Sci. Adv.* **1**, e1500087 (2015).

13. F. Bouchard, R. Fickler, R. W. Boyd, E. Karimi, High-dimensional quantum cloning and applications to quantum hacking. *Sci. Adv.* **3**, e1601915 (2017).

14. A. M. Yao, M. J. Padgett, Orbital angular momentum: Origins, behavior and applications. *Adv. Opt. Photonics* **3**, 161–204 (2011).

15. A. Forbes, L. Mkhumbuza, L. Feng, Orbital angular momentum lasers. *Nat. Rev. Phys.* **6**, 352–364 (2024).

16. A. Kavokin, G. Malpuech, M. Glazov, Optical spin Hall effect. *Phys. Rev. Lett.* **95**, 136601 (2005).

17. K. Y. Bliokh, F. J. Rodríguez-Fortuño, F. Nori, A. V. Zayats, Spin–orbit interactions of light. *Nat. Photonics* **9**, 796–808 (2015).

18. V. Sala, D. Solnyshkov, I. Carusotto, T. Jacqmin, A. Lemaître, H. Terças, A. Nalitov, M. Abbarchi, E. Galopin, I. Sagnes, J. Bloch, G. Malpuech, A. Amo, Spin-orbit coupling for photons and polaritons in microstructures. *Phys. Rev. X* **5**, 011034 (2015).

19. N. Carlon Zambon, P. St-Jean, M. Milićević, A. Lemaître, A. Harouri, L. Le Gratiet, O. Bleu, D. Solnyshkov, G. Malpuech, I. Sagnes, S. Ravets, A. Amo, Optically controlling the emission chirality of microlasers. *Nat. Photonics* **13**, 283–288 (2019).

20. R. Barczyk, L. Kuipers, E. Verhagen, Observation of landau levels and chiral edge states in photonic crystals through pseudomagnetic fields induced by synthetic strain. *Nat. Photonics* **18**, 574–579 (2024).

21. X. Zhang, F. Zangeneh-Nejad, Z.-G. Chen, M.-H. Lu, J. Christensen, A second wave of topological phenomena in photonics and acoustics. *Nature* **618**, 687–697 (2023).

22. P. Q. Jin, Y. Q. Li, F. C. Zhang, SU(2)xU(1) unified theory for charge, orbit and spin currents. *J. Phys. A. Math. Gen.* **39**, 11129 (2006).

23. S. Sugawa, F. Salces-Carcoba, A. R. Perry, Y. Yue, I. Spielman, Second Chern number of a quantum-simulated non-Abelian Yang monopole. *Science* **360**, 1429–1434 (2018).

24. D. Cheng, K. Wang, C. Roques-Carmes, E. Lustig, O. Y. Long, H. Wang, S. Fan, Non-Abelian lattice gauge fields in photonic synthetic frequency dimensions. *Nature* **637**, 52–56 (2025).

25. B. T. T. Wong, S. Yang, Z. Pang, Y. Yang, Synthetic non-Abelian electric fields and spin-orbit coupling in photonic synthetic dimensions. *Phys. Rev. Lett.* **134**, 163803 (2025).

26. L. Polimeno, A. Fieramosca, G. Lerario, L. De Marco, M. De Giorgi, D. Ballarini, L. Dominici, V. Ardizzone, M. Pugliese, C. Prontera, V. Maiorano, G. Gigli, C. Leblanc, G. Malpuech, D. D. Solnyshkov, D. Sanvitto, Experimental investigation of a non-Abelian gauge field in 2D perovskite photonic platform. *Optica* **8**, 1442–1447 (2021).

27. K. Rechcińska, M. Król, R. Mazur, P. Morawiak, R. Mirek, K. Łempicka, W. Bardyszewski, M. Matuszewski, P. Kula, W. Piecek, P. G. Lagoudakis, B. Piętka, J. Szczytko, Engineering spin-orbit synthetic Hamiltonians in liquid-crystal optical cavities. *Science* **366**, 727–730 (2019).

28. M. Muszyński, P. Oliwa, P. Kokhanchik, P. Kapuściński, E. Oton, R. Mazur, P. Morawiak, W. Piecek, P. Kula, W. Bardyszewski, B. Piętka, D. Bobylev, D. Solnyshkov, G. Malpuech, J. Szczytko, Electrically tunable spin-orbit coupled photonic lattice in a liquid crystal microcavity. *Laser Photonics Rev.* **19**, 2400794 (2024).

29. N. D. Mermin, The topological theory of defects in ordered media. *Rev. Mod. Phys.* **51**, 591–648 (1979).

30. J.-S. Wu, I. I. Smalyukh, Hopfions, heliknotons, skyrmions, torons and both abelian and nonabelian vortices in chiral liquid crystals. *Liq. Cryst. Rev.* **10**, 34–68 (2022).

31. L. Marrucci, C. Manzo, D. Paparo, Optical spin-to-orbital angular momentum conversion in inhomogeneous anisotropic media. *Phys. Rev. Lett.* **96**, 163905 (2006).

32. J. Kobashi, H. Yoshida, M. Ozaki, Polychromatic optical vortex generation from patterned cholesteric liquid crystals. *Phys. Rev. Lett.* **116**, 253903 (2016).

33. E. Brasselet, N. Murazawa, H. Misawa, S. Juodkazis, Optical vortices from liquid crystal droplets. *Phys. Rev. Lett.* **103**, 103903 (2009).

34. Q. Zhan, Cylindrical vector beams: From mathematical concepts to applications. *Adv. Opt. Photonics* **1**, 1–57 (2009).

35. M. Papič, U. Mur, K. P. Zuhail, M. Ravnik, I. Muševič, M. Humar, Topological liquid crystal superstructures as structured light lasers. *Proc. Natl. Acad. Sci. U.S.A.* **118**, e2110839118 (2021).

36. B. A. Bernevig, T. L. Hughes, S.-C. Zhang, Quantum spin Hall effect and topological phase transition in HgTe quantum wells. *Science* **314**, 1757–1761 (2006).

37. M. Z. Hasan, C. L. Kane, Colloquium: Topological insulators. *Rev. Mod. Phys.* **82**, 3045–3067 (2010).

38. R. Jackiw, C. Rebbi, Solitons with fermion number 1/2. *Phys. Rev. D* **13**, 3398 (1976).

39. A. Varanytsia, G. Posnjak, U. Mur, V. Joshi, K. Darrah, I. Muševič, S. Čopar, L.-C. Chien, Topology-commanded optical properties of bistable electric-field-induced torons in cholesteric bubble domains. *Sci. Rep.* **7**, 16149 (2017).

40. P. J. Ackerman, Z. Qi, I. I. Smalyukh, Optical generation of crystalline, quasicrystalline, and arbitrary arrays of torons in confined cholesteric liquid crystals for patterning of optical vortices in laser beams. *Phys. Rev. E Stat. Nonlinear Soft Matter Phys.* **86**, 021703 (2012).

41. Y. Shen, M. Qaiser, I. Dierking, Temperature reconfigurable skyrmionic solitons in cholesteric liquid crystals. *Soft Matter* **19**, 9325 (2023).

42. P. J. Ackerman, I. I. Smalyukh, Diversity of knot solitons in liquid crystals manifested by linking of preimages in torons and hopfions. *Phys. Rev. X* **7**, 011006 (2017).

43. I. V. Tokatly, Equilibrium spin currents: Non-Abelian gauge invariance and color diamagnetism in condensed matter. *Phys. Rev. Lett.* **101**, 106601 (2008).

44. H. Terças, H. Flayac, D. D. Solnyshkov, G. Malpuech, Non-abelian gauge fields in photonic cavities and photonic superfluids. *Phys. Rev. Lett.* **112**, 066402 (2014).

45. Y. Yang, B. Yang, G. Ma, J. Li, S. Zhang, C. Chan, Non-Abelian physics in light and sound. *Science* **383**, eadf9621 (2024).

46. K. Łempicka-Mirek, M. Król, H. Sigurdsson, A. Wincukiewicz, P. Morawiak, R. Mazur, M. Muszyński, W. Piecek, P. Kula, T. Stefaniuk, M. Kamińska, L. De Marco, P. G. Lagoudakis, D. Ballarini, D. Sanvitto, J. Szczytko, B. Piętka, Electrically tunable berry curvature an strong light-matter coupling in liquid crystal microcavities with 2D perovskite. *Sci. Adv.* **8**, eabq7533 (2022).

47. C. Schneider, K. Winkler, M. D. Fraser, M. Kamp, Y. Yamamoto, E. Ostrovskaya, S. Höfling, Exciton-polariton trapping and potential landscape engineering. *Rep. Prog. Phys.* **80**, 016503 (2016).

48. T. Jacqmin, I. Carusotto, I. Sagnes, M. Abbarchi, D. Solnyshkov, G. Malpuech, E. Galopin, A. Lemaître, J. Bloch, A. Amo, Direct observation of Dirac cones and a flatband in a honeycomb lattice for polaritons. *Phys. Rev. Lett.* **112**, 116402 (2014).

49. R. Mirek, P. Kokhanchik, D. Urbonas, I. Georgakilas, M. Muszyński, P. Kapuściński, P. Oliwa, B. Piętka, J. Szczytko, M. Forster, U. Scherf, P. Morawiak, W. Piecek, P. Kula, D. Solnyshkov, G. Malpuech, R. F. Mahrt, T. Stöferle, In situ tunneling control in photonic potentials by Rashba–Dresselhaus spin–orbit coupling. *Optica* **12**, 1548–1552 (2025).
